# Comparison of macular buckling and vitrectomy for the treatment of macular schisis and associated macular detachment in high myopia: a randomized clinical trial

**DOI:** 10.1111/aos.14260

**Published:** 2019-11-17

**Authors:** Bingqian Liu, Shida Chen, Yonghao Li, Ping Lian, Xiujuan Zhao, Xiling Yu, Tao Li, Chenjin Jin, Xiaoling Liang, Suber S. Huang, Lin Lu

**Affiliations:** ^1^ State Key Laboratory of Ophthalmology Zhongshan Ophthalmic Center Sun Yat‐Sen University Guangzhou China; ^2^ Retina Center of Ohio South Euclid Ohio USA

**Keywords:** high myopia, macular buckling, macular detachment, macular schisis, pars plana vitrectomy

## Abstract

**Objective:**

To compare the efficacy and safety of macular buckling and vitrectomy for myopic traction maculopathy showing macular schisis (MS) and associated macular detachment (MD) but without full‐thickness macular hole (FTMH).

**Design:**

Prospective, randomized, parallel, open‐label study.

**Methods:**

Patients were randomly assigned to either buckling or vitrectomy group. Macular buckling and intravitreal C3F8 gas injection were performed in the buckling group. Small gauge vitrectomy, internal limiting membrane peeling (ILMP) and C3F8 gas tamponade were performed in the vitrectomy group. The patients were followed for 12 months.

**Main outcome measures:**

Best‐corrected visual acuity (BCVA) at 12 months.

**Results:**

A total of 85 patients were randomized, 80 eyes were included (41 receiving buckling, 39 received vitrectomy), and 78 patients completed the study. There were less eyes determined as surgical failure and required a second surgery in the buckling group than vitrectomy the group (2.4% versus 18.4%, p = 0.021). After surgery, macular buckling achieved more improvement in BCVA (+21.7 versus +4.5 Early Treatment Diabetic Retinopathy Study (ETDRS) letters, p = 0.002). FTMH development was observed in only 1 (2.4%) eye, after removing of the implant due to recurrent conjunctival erosion, in the buckling group and 10 (26.3%) eyes (seven with‐, three without MD) in the vitrectomy group (p < 0.001). More eyes developed cataracts in the vitrectomy group than did in the buckling group (28.9% versus 7.5%, p = 0.014). Macular buckling‐associated strabismus (esotropia), binocular diplopia and implant exposure were observed in limited cases.

**Conclusions and relevance:**

Macular buckling is superior to vitrectomy with ILM peeling plus gas injection for surgical treatment of MS and associated MD in high myopia.

## Introduction

High myopia, a common cause of vision loss, afflicted about 163 million people (2.7% of the world population) in 2000. The prevalence of high myopia is increasing globally, as it is estimated that there will be almost 1 billion people (9.8% of the world population) with high myopia in 2050 (Holden et al. 2016). High myopia is associated with a series of pathologic complications, including posterior staphyloma, myopic macular schisis (MMS), epiretinal membrane, chorioretinal dystrophy, macular hole and retinal detachment (Baba et al. 2003; Chang et al. 2013; Henaine‐Berra et al. 2013).

The most common myopic traction maculopathy (MTM) of high myopia on optical coherence tomography (OCT) imaging is MMS (Panozzo & Mercanti 2004). Myopic macular schisis associated with macular detachment (MD) has been reported to be the most common pathway leading to severe vision loss (Sun et al. 2010; Theodossiadis et al. 2014). Currently, interventions for MMS include pars plana vitrectomy (PPV) and macular buckling. Pars plana vitrectomy (PPV) is effective for releasing the inner traction of MMS, prompting recovery from MD and improving vision postoperatively (Kanda et al. 2003; Ikuno et al. 2004; Kim et al. 2012). However, the traction of the posterior staphyloma remains unsolved by this method, and postoperative development of macular hole after internal limiting membrane peeling (ILMP) for MMS ranges from to 3% to 27% in highly myopic eyes (Ikuno & Tano 2006; Gaucher et al. 2007; Gao et al. 2013).

Macular buckling can relieve the traction of the posterior staphyloma and result in anatomic improvement and visual elevation(Burés‐Jelstrup et al. 2014; Zhu et al. 2016; Wu et al. 2017) In our previous study, we found macular buckling as primary procedure could improve anatomic disorder and visual decrease caused by MMS (Liu et al. 2016). Recent comparative studies have shown that macular buckling achieved better visual improvement and anatomic recovery than vitrectomy in highly myopic eyes with macular hole‐related retinal detachment (Ripandelli et al. 2001; Ando et al. 2007; Parolini et al. 2013; Alkabes et al. 2014). However, all the comparative studies were retrospective.

This trial aimed to compare the effects and safety of modified T‐type macular buckling and gas injection versus vitrectomy, ILMP and gas tamponade for the treatment of highly myopic eyes with MMS and accompanying MD. We tested the hypothesis that macular buckling would be associated with better vision improvement and less need for secondary surgery compared with vitrectomy.

## Methods

### Trial design

This is a single site, randomized, parallel, open‐label trial. Patients with high myopia and who showed MMS were recruited between April 2015 and October 2017 at the Zhongshan Ophthalmic Center in Guangzhou, China. Patients who met the inclusion criteria were randomly assigned to the buckling or vitrectomy group. The study was approved by the ethics committee of the Zhongshan Ophthalmic Center and conducted in accordance with applicable local regulations and with the principles described in the Declaration of Helsinki. Written informed consent was obtained from all patients. This study was registered on the website ClinicalTrials.gov with the ID NCT03023800.

### Participants

**Figure 1:**
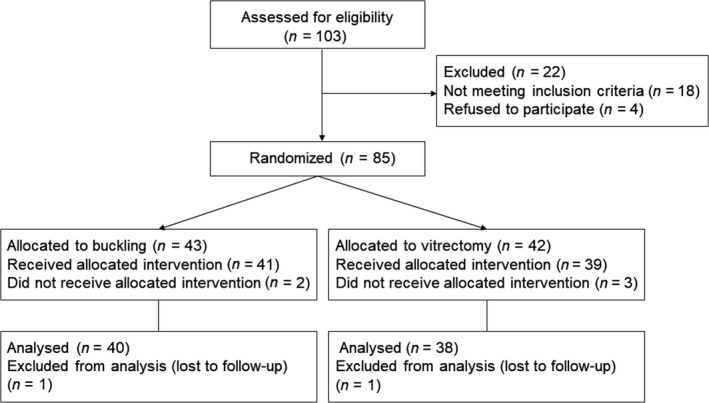
Consort diagram.

A total of 103 patients with high myopia and showing MTM on OCT imaging were screened for this study. Eighteen patients were excluded because of not meeting the eligibility criteria. Finally, 85 patients were enrolled for randomization (Fig. 1).

The eligibility criteria for the study included the following: highly myopic patients aged from 18 to 70 years; axial length ≥26.5 mm or refractive error (spherical equivalent) ≥8.0 dioptre; presence of MS and concurrent MD on OCT (lamellar macular holes might be present); and evidence of posterior staphyloma involving the macular area.

The exclusion criteria included the following: presence of full‐thickness macular hole (FTMH); presence of severe macular scar; MD that extends to peripheral retina (i.e. extension beyond the major vascular arcades in more than one quadrant); a history of vitrectomy or scleral buckling; presence of intraocular active haemorrhage or inflammation; and presence of any media opacity that precluded imaging or clinical evaluation of the macula.

### Randomization and masking

Using a binary random number generator, patients were randomized in a 1:1 ratio to undergo either macular buckling or vitrectomy. The randomized intervention was placed in a sealed envelope that was opened before the surgery. Given that the patients needed to know the detailed procedure and potential complications, it is not possible to mask both the participants and the doctors who performed the surgery. However, the technicians who performed best‐corrected visual acuity (BCVA) assessment, OCT and intraocular pressure (IOP) measurements were masked.

### Procedure

**Figure 2:**
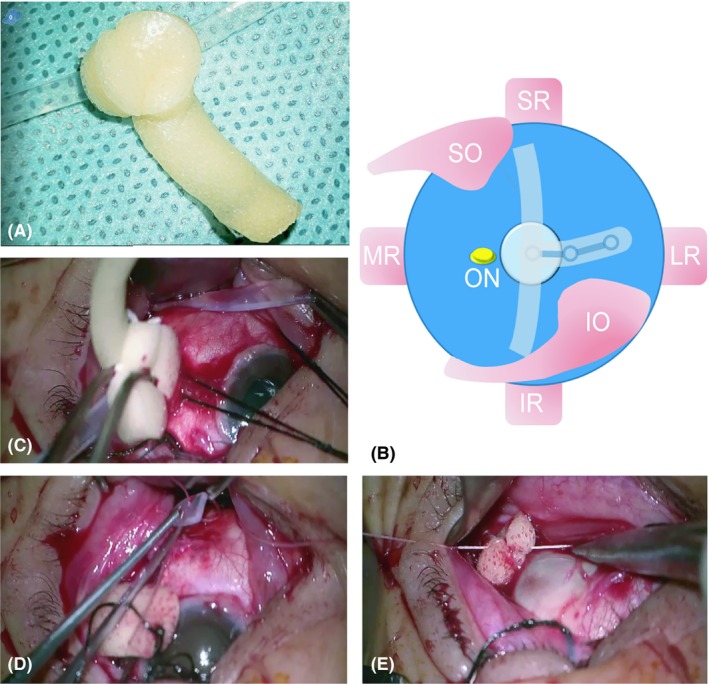
Photograph of implant and surgical procedure. (A) Modified T‐type macular buckling, which was made of silicone sponge, titanium plate (wrapped in the sponge), and an encircling silicone belt. (B) Schematic diagram of the buckling implant from a posterior view. The three arms of the buckle were placed underneath the rectus muscles. IO = inferior oblique, IR = inferior rectus, LR = lateral rectus, MR = medial rectus, ON = optic nerve, SO = superior oblique, SR = superior rectus. (C) Inserting the buckle implant into the sub‐Tenon's space, until reaching the posterior pole. (D) Suturing the inferior belt end on episclera around equator level underneath the inferior rectus, with the same procedure for the superior arm end fixation. (E) Suture fixation of the titanium strengthened arm end behind the equator level underneath the lateral rectus.

In macular buckling group, surgical procedures of macular buckling, drainage of aqueous fluid and C3F8 gas injection into the vitreous cavity through pars plana were performed. In brief, a T‐type buckle implant was made manually using silicone sponge strengthened with a titanium plate and a #240 encircling silicone belt (Fig. 2A). A 360° peritomy of the conjunctiva and the Tenon's capsule was performed around the limbus. The lateral, inferior and superior rectus muscles were isolated using 4‐0 silk threads to facilitate the mobility of the eye. The buckle plate was inserted to the posterior pole, through Tenon's peritomy by handling the titanium arm. The inferior silicone arm crossed the lateral rectus, inferior oblique and inferior rectus; the superior arm crossed the superior rectus; and the titanium was left parallel to the lateral rectus. The ends of the silicone belt were suture‐fixated on the episclera, 12–14 mm away from limbus, at the nasal side of the superior/inferior rectus, using a 5‐0 nonabsorbable thread (Ethicon, Johnson & Johnson, Shanghai, China). Then, a paracentesis at the limbus was performed to lower the IOP, using a 30‐gauge needle. The titanium arm end was fixed beside the lateral rectus muscle at the superior temporal quadrant, 16–21 mm away from the limbus. The fundus was monitored using a binocular ophthalmoscope to ensure the final position of the buckle. Adjusting the tension of the silicone band or the suture position of the titanium arm end was performed when necessary (Fig. 2B–E). Finally, 0.2–0.3 ml of C3F8 gas was injected into the vitreous chamber through the pars plana. We used gas injection in cases of macular buckling to balance the two groups; as in vitrectomy group, we used gas tamponade. After surgery, the patients were asked to maintain a facedown position for 1 week.

In the vitrectomy group, procedures of small gauge vitrectomy, ILMP and C3F8 gas tamponade were performed. In brief, transconjunctival 23‐ or 25‐gauge vitrectomy through pars plana was performed regularly. After removing the core and posterior hyaloid, the internal limiting membrane was stained with indocyanine green and peel completely including foveal area. The range of ILMP covered the whole macular area and crossed the main vascular arcade. Then, air–fluid exchange was performed, and the vitreous cavity was filled with C3F8 gas (14%). After surgery, the patients were asked to maintain a facedown position for at least 1 week.

### Outcome assessment

The included patients were evaluated at postoperative day 1, week 1, month 1, month 3, month 6, month 9 and month 12. A 2‐week window was allowed for the follow‐up visits. Outcomes measurements included FTMH development, BCVA change, OCT and surgical failure rate. Surgery‐associated complications were recorded.

All patients underwent BCVA test, axial length measurement with an IOLMaster, ocular motility assessment, IOP measurement, slit‐lamp biomicroscopy, fundus photography and indirect ophthalmoscopy. Optical coherence tomography (OCT) images were obtained using a SPECTRALIS OCT (Heidelberg Engineering, Heidelberg, Germany). High‐resolution horizontal/vertical cross‐hair scans (centred on the fovea) and volume OCT B‐scans (over a 30 × 25–degree field of view) were obtained.

Surgical failure was determined as the presence of postoperative FTMH and MD, which required a second surgery as judged by retinal surgeons. For eyes determined as surgical failure, a secondary operation of vitrectomy, air‐fluid exchange, laser photocoagulation of the margin of the macular hole and C3F8 gas (14%) tamponade was performed to reattach the retina. The final BCVA of the eyes on which a second surgery was performed was included for data analysis.

#### Data management

Each examination had a standard operation procedure (SOP) and a standard protocol for performing all data collection and follow‐up. Every staff member was trained before engaging this study. The investigator ensured that each study member followed the SOP to obtain reliable results. The data were well kept and backed up at each study visit.

### Sample size

The sample size was calculated based on the published surgical failure rate for the highly myopic with MTM. Surgical failure is defined as the presence of postoperative FTMH and MD, which require a second surgery (Gaucher et al. 2007; Gao et al. 2013). It is assumed that the 12‐month surgical failure rate would be 2.5% after receiving macular buckling and 25% after receiving vitrectomy, with 80% statistical power and a two‐sided test. Thus, to allow for a 10% loss during follow‐up, a total of 80 participants were required in this study, with 40 patients in each arm calculated using G‐Power 3.1.9.2 (Program written by Franz Faul, Universität Kiel, Kiel, Germany).

### Statistical analysis

Data were processed and analysed using spss for Windows (Version 13.0; SPSS, Chicago, IL, USA). All data are presented as mean ± standard deviation. Comparison of the normally distributed continuous variables between the two groups was determined using two independent *t*‐tests, while a Mann–Whitney *U*‐test was used for non‐normally distributed continuous variables. Qualitative data were assessed individually using chi‐square tests. For all tests, p < 0.05 was considered significant.

## Results

### Baseline characteristics

A total of 85 eyes from 85 patients were enrolled and randomly assigned to one of the two groups. The types of MTM on OCT were balanced well in both groups. Eighty patients were finally included, with 41 eyes receiving buckling and 39 eyes receiving vitrectomy. One patient from each group was not included in the analysis because of a loss of follow‐up after surgery; as such, 78 of the 80 patients completed the whole study. The two groups were well matched with respect to baseline ocular characteristics and demographics (Table 1), although the patients in the buckling group were slightly younger than those in the vitrectomy group (49.3 ± 10.9 versus 53.7 ± 9.4, respectively; p = 0.196).

**Table 1:** Demographic and baseline characteristics.

Characteristics	Buckling (*N* = 40)	Vitrectomy (*N* = 38)	p value
Age	49.3 (10.9)	53.7 (9.4)	0.196
Male sex‐No. (Female)	21 (19)	14 (24)	0.165
IOP (mmHg)	14.7 (4.0)	14.2 (2.9)	0.984
AL(mm)	29.6 (1.8)	29.5 (1.8)	0.676
SE (Dioptre)	−13.6 (5.0)	−14.6 (5.7)	0.386
BCVA (ETDRS letters)	22.4 (19.3)	29.1 (20.5)	0.208

AL = axial length, BCVA = best‐corrected visual acuity, ETDRS = early treatment diabetic retinopathy study, IOP = intraocular pressure, SE = spherical equivalent.

### Efficacy

#### Surgical failure

**Figure 3:**
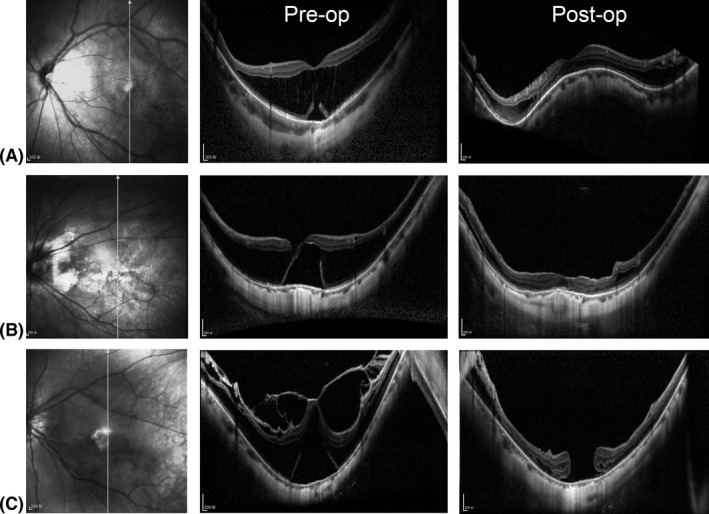
Typical optical coherence tomography images of eyes received macular buckling or vitrectomy. (A) Typical case of macular buckling group. (B, C) Typical cases of vitrectomy group. Left lane: infrared image of macular area. Middle lane: pre‐operation optical coherence tomography images. Right lane: postoperation images at month 12.

Within the follow‐up period of 12 months, the buckling group had fewer surgical failures than did the vitrectomy group (2.5% versus 18.4%, respectively; p = 0.021). Typical OCT images of eyes received macular buckling or vitrectomy are shown in Fig. 3. One case of macular hole and macular detachment, determined as surgical failure, in the buckling group occurred at 3 months after removing of the implant due to recurrent conjunctival erosion. We found age, preoperative axial length, BCVA, or refractive error was not associated with surgical failure. Preoperative OCT showing lamellar macular hole or severe traction on surface of thin fovea was associated with development of FTMH and surgical failure after vitrectomy and ILMP (Fig. 3C). After vitrectomy, three eyes developed FTMH but without MD, which were not judged as surgical failure. In total, FTMH development was observed in 10 (26.3%) eyes (three without MD) in the vitrectomy group and 1 (2.4%) in the buckling group (p < 0.001). All the eyes developed FTMH within 3 months after surgery. All the eyes determined as surgical failure, a secondary surgery of macular hole margin laser photocoagulation and gas tamponade resulted in anatomical success.

Patients who underwent macular buckling had subfoveal liquid that remained longer than those who underwent vitrectomy; about 57.5% of patients who received buckling had subfoveal liquid that lasted over 3 months, while only 36.8% of patients in the vitrectomy group had subfoveal liquid past 3 months (p = 0.068). Fortunately, all the subfoveal liquid that remained after surgery was relieved within 12 months (Table 2).

**Table 2:** Change of main outcomes at 12 months.

Measurements	Buckling (*N* = 40)	Vitrectomy (*N* = 38)	p value
BCVA changes*(SD)	+21.7 (18.7)	+4.5 (22.6)	0.002
Subfoveal liquid sustained >3 months (%)	23 (57.5%)	14 (36.8%)	0.068
Surgery failure No. (%)	1 (2.5%)	7 (18.4%)	0.021

* BCVA was recorded by the No. of total ETDR letters, data were presented as mean (SD).

BCVA = best‐corrected visual acuity, FTMH = full‐thickness macular hole, SD = standard deviation.

#### Best‐corrected visual acuity

**Figure 4:**
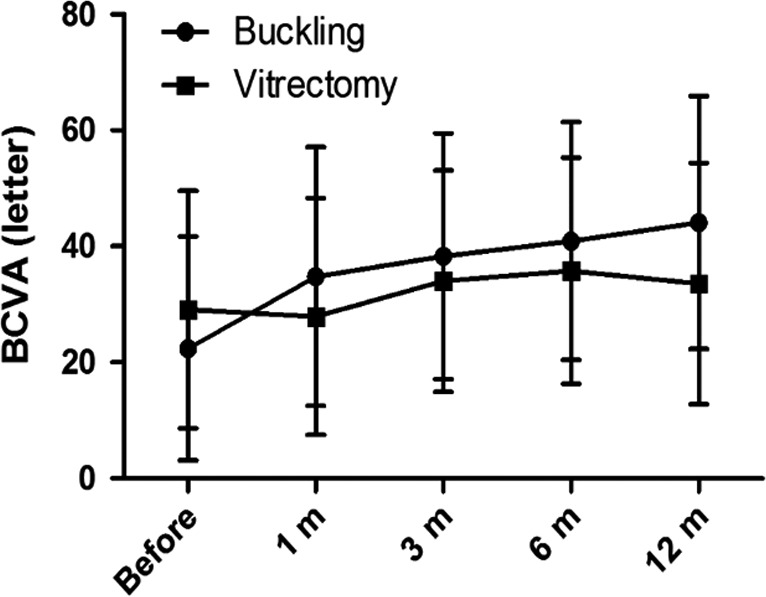
Changes of best‐corrected visual acuity (BCVA) after surgery. The BCVA (early treatment diabetic retinopathy study letter) of baseline and at month 1, month 3, month 6 and month 12 after surgical intervention is shown. Note: failure cases included.

Macular buckling was superior in terms of BCVA when compared with vitrectomy from the baseline to month 12 (+21.7 ± 18.7 versus +4.5 ± 22.6, respectively; p = 0.002; Table 2). Overall, the BCVA improved from the baseline (22.4 ± 19.3 letters) to month 12 (44.1 ± 21.8 letters) in the macular buckling group, and from the baseline (29.1 ± 20.5 letters) to month 12 (33.6 ± 20.8 letters) in the vitrectomy group. In the macular buckling group, the BCVA of the patients was improved at month 1 and then gradually improve more at follow‐ups over the following 12 months. However, in vitrectomy, the BCVA improved slightly and then stayed stable for the following 12 months (Fig. 4). Overall, in the buckling group, the BCVA improved in 34 of 40 eyes (85%); 62.5% of patients improved by more than 15 letters; two patients got worse; and four remained unchanged. In the PPV group, the BCVA improved in 22 of 38 eyes (57.9%); 36.8% patients improved by more than 15 letters; 11 patients getting worse; and 5 remained unchanged (Table 3). In vitrectomy group, the mean BCVA with failure cases excluded was a little better than that with failure cases included, but the final vision improvement was still worse than buckling group.

**Table 3:** Mean change of best‐corrected visual acuity (ETDRS letters) after surgery.

Intervention		1 month	3 months	6 months	12 months
Failure cases included	Buckling	+12.4 (16.0)	+15.9 (17.4)	+18.6 (18.5)	+21.7 (18.7)
	Vitrectomy	−1.3 (19.9)	+4.9 (18.1)	+6.7 (19.9)	+4.5 (22.6)
Failure cases excluded	Buckling	+11.9 (15.9)	+16.1 (17.6)	+18.6 (18.7)	+22.3 (18.5)
	Vitrectomy	−0.6 (20.4)	+4.1 (19.1)	+6.5 (19.0)	+6.6 (22.5)

ETDRS = early treatment diabetic retinopathy study.

#### Complications

After buckling, patients tended to have a higher IOP within 1 month (mean 22.2 ± 8.1 mmHg at month 1); in all cases, this could be controlled using pressure‐lowering agents (mean 16.8 ± 5.3 mmHg at month 3). After vitrectomy, patients had a stable IOP from the baseline to month 12 (Table 4)**.** Surgically induced esotropia (1/40, 2.5%), binocular diplopia (2/40, 5%), implant exposure (1/40, 2.5%) and vitreous haemorrhage (1/40, 2.5%) were found in limited cases of the buckling group. There were more patients in the vitrectomy group who developed mild to severe cataracts at the last follow‐up visit than there were in the buckling group (Table 5). Metamorphopsia was a common symptom before surgery, but this complication was gradually resolved over time, as shown in Table 6. Most patients reported that the symptom of metamorphopsia significantly decreased from month 3 after surgery in the buckling group.

**Table 4:** Change of intraocular pressure (mmHg) after surgery.

Intervention	Before surgery	1 day	1 month	12 months
Buckling	14.7 (4.0)	22.2 (8.1)^a^	16.8 (5.3)	15.1 (3.9)
Vitrectomy	14.2 (2.9)	11.9 (5.3)	16.0 (4.9)	15.2 (2.6)

a Compared to baseline, p < 0.001.

**Table 5:** Complications.

Event	Macular buckling (*n* = 40)	Vitrectomy (*n* = 38)
Transient ocular hypertension	15 (37.5%)	9 (23.7%)
Vitreous haemorrhage	1 (2.5%)	0
Binocular diplopia	2 (5%)	NA
Esotropia	1 (2.5%)	NA
Implant exposure	1 (2.5%)	NA
Mild to severe Cataract	3 (7.5%)	11 (28.9%)
Development of FTMH	0 (0.0%)	10 (26.3%)

FTMH = full‐thickness macula hole, NA = not applicant.

**Table 6:** Symptom of metamorphosia.

Intervention	Before surgery (%)	Month 1 (%)	Month 3 (%)	Month 12 (%)
Buckling (*n* = 40)	25 (62.5)	21 (52.5)	13 (32.5)	3 (7.5)
Vitrectomy (*n* = 38)	22 (57.9)	11 (28.9)	7 (18.4)	5 (13.2)

## Discussion

This study explored the effects and safety of modified T‐shape macular buckling in comparison with PPV on MTM of MS with concurrent MD in high myopia. Overall, macular buckling provided better postoperative improvement of BCVA. The macular buckling group also showed fewer surgical failures than did the vitrectomy group. As such, it was an effective intervention for MTM in high myopia.

Myopic traction maculopathy (MTM) is considered to be a result of the opposite tractions of the posterior staphyloma and vitreoretinal interface(Takano & Kishi 1999). Releasing either of these two tractions has been reported to be effective in improving anatomic disorder and visual decrease. Vitrectomy and ILMP can release the inner traction of MTM(Ikuno et al. 2008); however, the pathologic bulging and stretching of the posterior sclera remain unsolved by this method, especially in eyes with a pronounced posterior staphyloma. Macular buckling may be more suitable for overcoming the posterior bulging of the eyeball (Theodossiadis & Theodossiadis 2005). The surgical failure rate in the macular buckling group was much lower than in the vitrectomy group in our study, and this result is consistent with other studies (Parolini et al. 2013, 2015; Cacciamani et al. 2016; Wu et al. 2017). Ando et al. (2007) retrospectively analysed 58 highly myopic eyes that underwent macular buckling or PPV, and they found that the retinal re‐attachment rate was 93.3% after primary macular buckling and 50% after primary PPV. Parolini et al. (2015) found that combined vitrectomy and buckling took more surgical time and led to more complications and therefore suggested that macular buckling alone should be the first choice for MTM. Our previous study using a three‐armed adjustable silicon capsule in highly myopic eyes with foveoschisis and found that none of the eight patients enrolled in the study required secondary surgery during the follow‐up time of about 1 year (Liu et al. 2016). In the present study, the need for a second surgery in the buckling group was only one of the 40 enrolled patients (2.5%). However, seven of the 38 enrolled patients (18.4%) in the vitrectomy group required a second surgery. Therefore, the initial use of macular buckling provided an effective intervention for highly myopic eyes with macular foveoschisis and MD.

Better postoperative BCVA improvements in the buckling group were observed in this study, which is consistent with previous studies (Ando et al. 2007). A retrospective trial demonstrated that there was limited BCVA improvement in the vitrectomy group but that there was significantly increased BCVA in the macular buckling group in highly myopic eyes with retinal detachment and FTMH (Ripandelli et al. 2001). Our previous study found that macular buckling significantly improved the BCVA followed for about 1 year (Liu et al. 2016). In the present study, in the buckling group, the BCVA improved by about 21 letters; in the vitrectomy group, the mean improvement of BCVA was about four letters, partially because there were more patients in which MD and FTMH occurred and more patients who developed mild to severe cataracts, which cause worsened BCVA.

Generally, there are two types of macular buckle material: absorbable material from donor tissue, such as sclera, and nonabsorbable material, such as silicone rubber or silicone sponge of various shapes (Devin et al. 2011; Bedda et al. 2015; Parolini et al. 2015). Our previous study used a three‐armed silicone capsule of a diameter of 8 mm, proved effective for controlling macular foveoschisis; however, this kind of buckle can cause a postoperative sub‐choroidal haemorrhage in 1/4 cases. Furthermore, the three‐armed capsule implant was soft, being made of silicone, and difficult to being inserted to accurate position, resulting in longer surgical time. In the present study, the three‐armed implant using a silicone sponge and titanium plate was easier to manipulate and associated with fewer complications.

Previously published model of silicone sponge and titanium stent was reported without IOP increase postoperatively (Parolini et al. 2015). In our series, transient IOP elevation was observed commonly after titanium–silicone sponge implantation, probably due to several reasons: the size of our implant was bigger; there was an additional encircling band in our implant; we injected C3F8, which might be also a contributor for the increased IOP; furthermore, subfoveal choroidal thickness was found transiently increased after macular buckling (unpublished observation), indicating the choroidal reflow of posterior pole was interfered to some extent.

During operation, we cannot determine the exact height of the buckling under indirect ophthalmoscope. But if the height was too high or too shallow, it could be observed and adjusted thereafter. In theory, it is ideal to use intraoperative OCT for judging the precise height of buckling. But it is difficult to find the focus of intraoperative OCT without intraocular illuminate, especially in eyes with long axil length. Furthermore, carefully controlled intraoperative IOP would help to stable the height of the buckle, as paracentesis is commonly performed to facilitate the implant insertion and avoid ocular hypertension‐related corneal oedema.

Metamorphopsia, a common symptom before surgery, gradually relieved 3 months after surgery in the majority of cases, possibly because the tension and height of the inward bulge from the buckle decreases gradually, to some extent; only very few patients remained symptomatic of metamorphopsia at 1 year after buckling. Indeed, one eye showed conjunctival erosion by the implant, two patients suffered from surgically induced binocular diplopia and one patient showed strabismus (esotropia). We think these rare but severe complications might be potentially prevented by reducing the length and thickness of the temporal arm of the buckling implant and adjusting the curved arc of the titanium plate to precisely fit the deformed eyeball. The most common complications after vitrectomy were the development of FTMH and cataracts, which happened in about 1/5 and 1/3 of patients, respectively.

However, this study also has some limitations. There were differences between the two groups concerning baseline age and BCVA, but the difference was of no statistical significance. And the axial length and the refractive error balanced very well at baseline. Participants, the operator and the fundus examiners were not possibly masked to the surgical treatments. The procedure of macular buckling can also be difficult if the eye is extremely myopic or the conjunctiva is scarring. Other indications of macular buckling were not included, such as a macular hole‐related retinal detachment, severe diffuse MS, or recurrent FTMH and MD after vitrectomy in highly myopic eyes. It is not possible to calculate the accuracy of buckle height by binocular ophthalmoscope.

In the macular buckling group, we used gas injection to balance the two groups. Gas injection might cause transient IOP elevation. Intraocular ophthalmitis or retinal injury might also be potential complications of intraocular gas injection procedure, although none of the complications was observed in our cases. In the vitrectomy group, preoperative OCT showing lamellar macular hole or severe traction on surface of thin fovea was associated with FTMH development and surgical failure after vitrectomy and ILMP, which suggest foveal sparing peeling technique might benefit the almost‐penetrating foveal tissue and avoid formation of FTMH. Furthermore, the need for long‐term follow‐up in terms of BCVA and macular structure is necessary and useful because of the natural progression of maculopathy in high myopia, a degenerative disease.

In conclusion, when comparing macular buckling and PPV, macular buckling achieved a higher surgical success rate, achieved more visual improvement and reduced majority FTMH development after surgery, when followed‐up with for 1 year. The safety profile, including the most common concerns following macular buckling, was found to be acceptable in this series, and further modification of the buckling implant might reduce the incidence of severe complications. Overall, macular buckling is an alternative option for the initial treatment of MTM in severe myopia.
